# The Key to the Future Lies in the Past: Insights from Grain Legume Domestication and Improvement Should Inform Future Breeding Strategies

**DOI:** 10.1093/pcp/pcac086

**Published:** 2022-06-17

**Authors:** Abhishek Bohra, Abha Tiwari, Parwinder Kaur, Showkat Ahmad Ganie, Ali Raza, Manish Roorkiwal, Reyazul Rouf Mir, Alisdair R Fernie, Petr Smýkal, Rajeev K Varshney

**Affiliations:** State Agricultural Biotechnology Centre, Centre for Crop and Food Innovation, Food Futures Institute, Murdoch University, 90 South Street, Murdoch, WA 6150, Australia; Crop Improvement Division, ICAR-Indian Institute of Pulses Research (ICAR-IIPR), Kalyanpur, Kanpur 208024, India; UWA School of Agriculture and Environment, The University of Western Australia, 35 Stirling Hwy, Crawley, WA 6009, Australia; Department of Biotechnology, Visva-Bharati, Santiniketan, Santiniketan Road, Bolpur 731235, India; Key Laboratory of Ministry of Education for Genetics, Breeding and Multiple Utilization of Crops, Center of Legume Crop Genetics and Systems Biology/College of Agriculture, Oil Crops Research Institute, Fujian Agriculture and Forestry University (FAFU), Fuzhou 350002, China; Khalifa Center for Genetic Engineering and Biotechnology (KCGEB), UAE University, Sheik Khalifa Bin Zayed Street, Al Ain, Abu Dhabi 15551, UAE; Division of Genetics & Plant Breeding, Faculty of Agriculture, SKUAST, Shalimar, Srinagar 190025, India; Department of Molecular Physiology, Max-Planck-Institute of Molecular Plant Physiology, Am Mühlenberg 1, Potsdam-Golm 14476, Germany; Department of Botany, Faculty of Sciences, Palacky University, Křížkovského 511/8, Olomouc 78371, Czech Republic; State Agricultural Biotechnology Centre, Centre for Crop and Food Innovation, Food Futures Institute, Murdoch University, 90 South Street, Murdoch, WA 6150, Australia

**Keywords:** Crop wild relatives, Diversification, Domestication, Genes, Grain legumes, Selective sweeps

## Abstract

Crop domestication is a co-evolutionary process that has rendered plants and animals significantly dependent on human interventions for survival and propagation. Grain legumes have played an important role in the development of Neolithic agriculture some 12,000 years ago. Despite being early companions of cereals in the origin and evolution of agriculture, the understanding of grain legume domestication has lagged behind that of cereals. Adapting plants for human use has resulted in distinct morpho-physiological changes between the wild ancestors and domesticates, and this distinction has been the focus of several studies aimed at understanding the domestication process and the genetic diversity bottlenecks created. Growing evidence from research on archeological remains, combined with genetic analysis and the geographical distribution of wild forms, has improved the resolution of the process of domestication, diversification and crop improvement. In this review, we summarize the significance of legume wild relatives as reservoirs of novel genetic variation for crop breeding programs. We describe key legume features, which evolved in response to anthropogenic activities. Here, we highlight how whole genome sequencing and incorporation of omics-level data have expanded our capacity to monitor the genetic changes accompanying these processes. Finally, we present our perspective on alternative routes centered on de novo domestication and re-domestication to impart significant agronomic advances of novel crops over existing commodities. A finely resolved domestication history of grain legumes will uncover future breeding targets to develop modern cultivars enriched with alleles that improve yield, quality and stress tolerance.

## Introduction

Domestication refers to an evolutionary process causing remarkable changes in plant morphology and physiology in response to the selection pressures created by anthropogenic activities (Zohary and Hopf 2000). The process of crop domestication was based on selection driven by human cultivation practices and agricultural environments as well as other population genetic processes such as a reduction in effective population size. Crop domestication likely began about 15,000 years ago in various parts of the world, with one of the oldest centers in the Middle East and Fertile Crescent.

Members of the Fabaceae family were domesticated as grain legumes in conjunction with the domestication of grasses for cereals (De Candolle 1883, Zohary and Hopf 2000, Abbo et al. 2012). Pea (*Pisum sativum* L.), faba bean (*Vicia faba* L.), lentil (*Lens culinaris* L.), grass pea (*Lathyrus sativus* L.) and chickpea (*Cicer arietinum* L.) are some of the world’s oldest domesticated crops and arose in the Fertile Crescent of Mesopotamian agriculture. These legumes accompanied cereal production and formed important dietary components of early civilizations in the Middle East and the Mediterranean (Zohary and Hopf 1973, Abbo et al. 2009, 2013). Similarly, soybean (*Glycine max* L.) and adzuki bean (*Vigna angularis* L.) accompanied cultivation with rice (*Oryza sativa* L.) in China, whereas domestication of common bean (*Phaseolus vulgaris* L.) and cowpea (*Vigna unguiculata* L.) occurred in Andes/Mesoamerica and sub-Saharan Africa, respectively. Other pulse crops such as pigeonpea (*Cajanus cajan* L.), mungbean (*Vigna radiata* L.) and urdbean (*Vigna mungo* L.) were domesticated in India (Fuller 2007).

Integrating evidence from archeological, genetic and recent omics studies has supported domestication as a multistage process (Fuller et al. 2014, Alseekh et al. 2021) that involves the origin of a crop as resulting from a continuous introgression of desirable genes, formation of the domesticated population and, finally, deliberate modern breeding methods. Morpho-physiological features became remarkably distinct between the wild ancestors and domesticated forms in response to the transitions from hunter-gatherer to systematic agriculture and further into large-scale crop production. Selection attributes according to the crop objective involve reduced seed and fruit dispersal, changes in the plant structure, plant phenology, seed dormancy, palatability or acquisition of modified fruit size and shape in vegetable crops (Meyer et al. 2012, Abbo et al. 2014, Fuller and Allaby 2018, Smýkal et al. 2018). These distinguishing crop features that enhanced reliance on human interventions are referred to as domestication syndrome traits (Harlan 1971). Genetic bottlenecks and founder events occurred during domestication due to shifts of the crops from their centers of origin. Therefore, beneficial genes including alleles imparting a broad spectrum of disease and pest resistance were lost during the process. In this context, crop wild relatives (CWRs) serve as a source of untapped genetic variation for use in breeding programs (Coyne et al. 2020, Bohra et al. 2022). Therefore, the identification and development of novel germplasm in breeding programs with better agronomic and resistance traits would buffer the effects of climatic changes on modern cultivars. The use of CWRs in commercial breeding, however, is not as easy in practice, due to the occurrence of linkage drag, cross incompatibilities and poor agronomic traits.

In this article, we discuss evidence from archeobotanical and plant genetics perspectives that have helped broaden our understanding of the domestication history of grain legume crops. We then describe how genome variation scanning on diverse accessions including CWRs and landraces has delimited the genomic regions associated with selection pressures due to ancient anthropogenic activities and systematic breeding. We also underscore the significance of grain legume CWRs as a source of novel genetic variations for breeding programs. We, therefore, highlight the main approaches used to map the domestication genes and explore the future opportunities and challenges that lie ahead in rapid manipulation of these genes by the use of mutation, introgression and gene-editing tools.

## Archeobotanical Evidence on Origin and Domestication of Grain Legumes

Archeobotany, the study of plant remains from sites of ancient location, provides data for understanding the initial evolution of domesticated plants. Based on the past history and archeological evidence, the Near East is considered as one of the primary domestication centers that might have affected other domestication centers worldwide. In light of carbonized remains from the Neolithic and Bronze/Iron ages, Fuller (2007) advocated the Middle East (10,000 years ago) or the Fertile Crescent as the center of origin for wild wheat (*Triticum* spp.) and barley (*Hordeum vulgare*), along with the grain legume crops such as pea and lentil. Wheat and barley were claimed to be originated in slightly drier, open parkland steppes along with wild grasses, while pulse crops including lentil, pea, chickpea and *Vicia* spp. originated in the clearings of nearby woodlands and rocky talus slopes (Zohary and Hopf 2000). When climatic conditions became favorable and nomad population started increasing during the Pre-Pottery Neolithic Period, an increased seed size and a softer seed coat were realized as more favorable features.

Based on the distribution of wild soybean (*Glycine soja*), it has been suggested that soybean domestication occurred simultaneously at multiple sites (Xu et al. 2002). Archeological records in the form of charred soybean seeds collected from Japan, China and Korea also support the hypothesis of multiple domestications in East Asia (Lee et al. 2011). The common bean has the longest domestication history of warm-season legumes, originating in parallel in two separate centers of domestication, one in the Andean mountains of South America, giving rise to the Andean genepool, and the other in the Central American highlands and lowlands, giving rise to the Mesoamerican genepool (Blair et al. 2012). Early archeological remains in the caves of the Ayacucho and Guerrero regions of Peru and Mexico, respectively, suggest that domestication could have occurred as early as 10,000 years ago. Four other related cultivated species in the genus *Phaseolus* were probably domesticated later on.

For pigeonpea, historical evidence suggests a relatively short cultivation history, starting in 400 BC to 300 AD. Until recently, the origin of pigeonpea was unclear, with some researchers suggesting an African origin, others India. Archeological, taxonomic and genomic studies suggest India as single center of origin (van der Maesen 1990, Varshney et al. 2017).

A range of important *Vigna* grain legumes was domesticated in Asia. These include mungbean and the urd bean from South and East Asia, respectively. Remains dating to 3500–3000 BC were found in archeological sites at Navdatoli in Central India (Jain and Mehra 1980). However, the domestication dates of other *Vigna* crops are largely unknown due to a lack of research, tropical climates and poor conditions for preservation of archeological remains. The cultivated *Arachis hypogaea* is derived from the spontaneous interspecific hybridization of two wild sympatric Arachis species, an event, which makes cultivated groundnut crop highly monomorphic.

Chickpea domestication went through a series of bottlenecks from its narrow origin as a southeast Anatolian winter annual (*Cicer reticulatum*) to its current status as a South Asian and spring-sown Mediterranean crop (Abbo et al. 2003, Varshney et al. 2021b). The earliest archeological remains of chickpea (10th millennium BP) were discovered within or close to the current distribution of *C. reticulatum* in southeast Anatolia (Von Wettberg et al. 2018). Thereafter, chickpea spread throughout the Eastern Mediterranean, presumably as a winter annual, like its wild progenitor, and was spread throughout the Mediterranean basin by the Greeks, Romans and Phoenicians. Following the early Mediterranean change from autumn- to spring-sowing and concomitant movement to warmer climates to the south and southeast (Africa and South Asia), chickpea has lost its vernalization requirement (Berger et al. 2011). A recent study based on 3,366 genomes suggested the migration of chickpea from its center of origin (Fertile Crescent) to South Asia and East Africa and to the Mediterranean region, the Black Sea and Central Asia (Varshney et al. 2021b).

Archeological evidence dates the existence of pea back to 10,000 BC in the Near East (Zohary and Hopf 2000) and Central Asia (Riehl et al. 2013). In Europe, pea has been cultivated since the Stone and Bronze Ages and in India from 200 BC (De Candolle 1883). Cultivation of pea spread from the Fertile Crescent westwards into ancient Greece and Rome, which further facilitated its spread to northern and western Europe. In parallel, pea cultivation moved eastward to Persia, India and China (Makasheva 1979). It was realized that a softer seed coat allowed faster imbibition and subsequent cooking and thus was preferred by nomads. Radiometric data were congruent of origin within the above-mentioned time period (Hopf 1983). More fossilized seeds that pre-date these are dated to 4800–4400 BC in the Nile area in Egypt and to 3800 BC in Upper Egypt (Zohary and Hopf 2000). The presence of smoother seed coat pea in 5850–5600 BC attests to the hypothesis of slower adaptive changes during domestication.

The oldest carbon fossil of lentils was found in 11,000 BC in Franchthi cave in Greece and remains from Tell Mureybit in Syria date back to 8000–7500 BC (Zohary and Hopf 1973, Hansen and Renfrew 1978, van Zeist and de Roller 1995). The small amount of lentil seeds obtained from (Jarmo) North Iraq, (Ali Kosh) Iran, (Can Hasan) Anatolia and Turkey, the places where early farming was practiced, were of a small size with a diameter in the range of nearly 2.5–3.0 mm (Zohary and Hopf 1973). The small seeds had a close association with the early cultivation of emmer wheat and barley. The seed remains of lentils (4.2 mm in diameter) at Yiftah-el (Israel) dating back to 6800 BC supported that the domestication had started (Zohary 1992). A change in the seed size was evident as an obvious development under domestication from the seed remains from 5500–5000 BC at Tepe Sabz, Iran, the oldest larger seed pointed toward the initiation of domestication (Helbaek 1969). Archeological data suggest that lentils were domesticated in Near East and later the crop spread in Cyprus in the Neolithic period, and during 3500–2800 BC, the crop remains were observed in samples collected from Prastio. At the same time, lentils diffused toward Southeastern Europe, and remains during the fifth millennium indicated domestication. The crop also moved toward the Nile valley in Neolithic times and from there it reached Ethiopia. Lentils moved eastwards in Georgia and adjacent to Bulgaria in the fifth and early fourth millennia and appeared in India and Pakistan around 2500–2000 BC (Sonnante et al. 2009). During the Bronze Age (3300–1200BC), the remains of peas and lentils in Europe were sparser than in the Neolithic time (Zohary and Hopf 1973).

The wild ancestor of cultivated faba bean does not exist in nature, and its origin remains still obscure. Faba bean remains have been found in archeological sites at Tell-el-Kerkh in northwest Syria, indicating that faba bean originated during the 10th millenium BC (Tanno and Willcox 2006). Recent investigations in the site of el-Wad (Mount Carmel, Israel) provide the first and, so far, only remains of the lost ancestor of faba bean (Caracuta et al. 2015, 2016).

Two lupin species, *Lupinus albus* L. and *Lupinus mutabilis* Sweet, were domesticated about 3000–4000 years ago in Egypt and the Andes, respectively. *Lupinus angustifolius* L. and *Lupinus luteus* L. were introduced into agriculture more recently in Northern Europe in the nineteenth century (Wolko et al. 2011).

## Genome Variation Maps in Grain Legumes Resolve Domestication and Improvement History

Analyzing genome-wide nucleotide variations based on biological status (wild, landraces and breeding lines) has become a standard approach to study crop domestication and evolution. Linkage disequilibrium (LD) encompasses alleles at two or more loci having non-random association, and patterns of LD decay reflect recombination rates in the population. For instance, resequencing of 302 soybean accessions including wild, landraces and breeding lines revealed the extent of LD decay (Zhou et al. 2015b), with wild showing the least LD extent (27 kb) as compared to landraces (83 kb) and breeding lines (133 kb). A similar pattern was reported in chickpea (Varshney et al. 2019) wherein the LD decay was slower in breeding lines (∼320 kb) than that of landraces (∼180 kb) and modern cultivars (∼190 kb). Examination of LD decay provides insights into the process of domestication as the positive selection of alleles results in extended LD blocks in modern cultivars as compared to CWRs (Varshney et al. 2019). To better resolve the domestication and improvement history, the crop geneticists analyze selection signals during transitions from wild to landraces (domestication) and from landraces to breeding lines/elite cultivars (improvement). Selection signals involve nucleotide variations in the genomic regions surrounding the causative alleles in response to natural or artificial selective pressures.

Genome sequencing of multiple accessions has revealed a loss of genetic diversity in breeding improved cultivars in different grain legume crops. For instance, the genome sequencing of pea and Pigeonpea revealed the most diverse single nucleotide polymorphisms (SNPs) in wild accessions in comparison to landraces and cultivars (Varshney et al. 2017, Kreplak et al. 2019). The genomic regions showing reduction of diversity (ROD) in wild vs. landraces and landraces vs. improved lines comparisons represent potential selection signatures (**Table 1**). The comparative analysis between wild and landraces and landraces and cultivars of pigeonpea revealed a total of 2,945 and 1,323 regions, respectively, with a maximum loss of diversity (Varshney et al. 2017). In chickpea, a total of 122 candidate genomic regions containing 204 genes showed potential signals of selection following domestication, i.e. diversification and improvement (Varshney et al. 2019). Recent genome sequencing studies assign a greater role to large structural variations (SVs) such as presence–absence variations (PAVs) and copy number variants (CNVs) in the crop domestication process, as exemplified by the presence of 32 CNVs and one PAV in the ROD region in common bean (Wu et al. 2020).

**Table 1:** Crucial genetic changes associated with domestication and improvement of grain legumes, elucidated by whole genome sequencing

Crop	Accessions	Genome-wide selection sweeps and candidate genes	References
Soybean	302 [62 wild (*G. soja*), 130 landraces and 110 improved cultivars]	Domestication and improvement created 121 and 109 selective sweeps, respectively	Zhou et al. (2015b)
		Domestication and improvement involved a total of 162 CNVs	
		Lower level of genetic diversity in cultivated soybeans compared to wild (cultivated: 1.89 × 10^−3^; wild: 2.97 × 10^−3^)	Lam et al. (2010)
Common bean	683 (529 landraces and 154 breeding lines)	A total of 268 candidate genes containing 2,205 SNPs across introns and exons were found in ROD regions (32 CNVs and 1 PAV) under selection	Wu et al. (2020)
		171 genomic regions were found to have been selected during breeding	
Chickpea	3,366 accessions (3,171 cultivated and 195 wild species)	Genomic regions that underwent selection during crucial transitions: wild to landraces (2,899; 42,148 kb) to landraces to breeding lines (191; 4,360 kb) and breeding lines to cultivars (14; 404 kb)	Varshney et al. (2021b)
		A set of 37 genes associated with adaptation to different environmental conditions	
	429 (268 landraces, 144 elite and breeding lines, 7 accessions of two wild species *Cicer echinospermum* and *C. reticulatum*)	204 genes underlying 122 candidate genomic regions were potentially selected during post-domestication diversification and breeding	Varshney et al. (2019)
	90 (60 improved lines, 25 landraces, 5 accessions of wild, *C. reticulatum* and *C. echinospermum*)	122 candidate genes selected during modern breeding efforts	Varshney et al. (2013)
		Reduction in nucleotide diversity from wild (3.80 per kb) to landraces (0.86 per kb) and breeding lines (0.84 per kb) implied to nearly 80% diversity loss during domestication	
Pea	494 (213 *P. fulvum*, 40 *P. abyssinicum*, 45 *P. sativum* and 196 *P. sativum* subsp*. elatius*) restriction-site associated DNA (RAD)-sequencing	Detection of genetic signature of selection two domestication events, one for *P. sativum* and second for *P. abyssinicum*	Hellwig et al. (2022)
Pigeonpea	292 (117 breeding lines, 166 landraces, 2 others and 7 accessions from three wild species, *Cajanus cajanifolius, Cajanus scarabaeoides* and *Cajanus platycarpus*)	Genomic regions associated with higher ROD values during transitions (wild to landraces: 2,945 and landrace to breeding lines: 1,323) might have experienced selection sweeps during domestication and improvement	Varshney et al. (2017)
		1,722 and 671 genomic regions were identified as regions with maximum diversity loss (ROD = 1) during domestication and breeding, respectively	

In chickpea, desi genotypes display higher SNPs, insertions or deletions (INDELs) and PAVs but lower CNVs as compared to kabuli (Varshney et al. 2019). These findings support the fact that wild *C. reticulatum* has early domesticated into desi type (common in South Asia and sub-Saharan Africa), whereas kabuli (common in West Asia and Mediterranean regions) were subsequently derived (Varma Penmetsa et al. 2016). The role of large SVs as an important source of variation has come to the fore with methodological improvements in the analysis of whole genome sequence data. In soybean *Rhg1*, a 31-kb CNV is a part of diversification trait conferring to multiple gene functions such as alpha soluble N-ethylmaleimide-sensitive factor attachment protein (SNAP) involved in snare membrane traffic, wound-inducible protein and an amino acid transporter (Cook et al. 2012). Similarly, in common bean, determinate growth conferred by the gene *PvTFL1Y* involves the deletion of 5840 bp and insertion of 4171 bp under domestication (Kwak et al. 2012). CNVs are found to be primarily associated with the diversification traits (Lye and Purugganan 2019).

The genomic evolution rate profiling (GERP) score reveals the genomic regions that remain evolutionarily constrained (GERP > 0) and indicates purifying selection, such as 29 Mb in chickpea (Varshney et al. 2021b) and 237.5 Mb in soybean (Kim et al. 2021). In chickpea, 37 non-synonymous SNPs representing deleterious mutations in 36 genes were fixed as these escaped purging through breeding (Varshney et al. 2021b). In soybean, domestication created 183 selective sweeps but reduced deleterious alleles by 7% in domesticates compared to the CWRs (Kim et al. 2021). As mentioned in the preceding sections, loss of genetic diversity during domestication and adaptation due to the bottleneck effect is common across domesticated crops. Genome variation analysis in 3,366 genomes of chickpea elucidated the occurrence of a strong bottleneck and a strong expansion around 10,000 and 400 years ago, respectively (Varshney et al. 2021b). Genomic regions influenced by selection were revealed during different transitions, i.e. wild to landraces (2,899; 42,148 kb), landraces to breeding line (191; 4,360 kb) and breeding line to elite cultivars (14; 404 kb) (Varshney et al. 2021b). This study also supports that a series of four bottlenecks occurred during the evolution and adaptation in chickpea (Abbo et al. 2003). Genetic clustering of the diverse accessions and the geographic distribution suggest diversification and the developmental processes (such as flower development *FLP, MYB12*) as the adaptive changes during shifting environments (Varshney et al. 2021b). In common bean, the occurrence of two separate domestication events was endorsed by genome sequencing (Schmutz et al. 2014). With a small founding population, the wild Andean gene pool diverged from the wild Mesoamerican gene pool 165,000 years ago, accompanied by a strong bottleneck that lasted 76,000 years (Schmutz et al. 2014). The study reinforced the independent evolution of the determinacy growth controlled by the *PvTFL1y* gene and the two gene pools. For domestication, the authors found 25 Mesoamerican and 13 Andean genes (such as *VRN1, VRN2, FRL1, TFL2, FRL1, TFL2* and *AGL42*) influencing the two key flowering-related genes *SOC1* and *FT*. The growing sequence data on whole genomes of diverse accessions will likely enrich the knowledge about the genetic underpinnings of the plant phenotypes that were changed during domestication and improvement of grain legume crops.

## Wild Relatives of Grain Legumes: A Rich Resource for Improving Crop Yield and Stress Response

The potential of CWRs and landraces has been historically recognized for supplying new genetic variation to plant breeding programs (Zhang et al. 2018, Coyne et al. 2020, Bohra et al. 2022). The valuable traits of legume CWRs and landraces for crop improvement encompass stress response and a high yield potential (**Supplementary Table S1**).

CWRs often encounter broad and contrasting environmental conditions across their native range. Over evolutionary time, these exert differential selection pressure that leads to the formation of specifically adapted ecotypes. Drought is one of the main abiotic stresses acting in the Mediterranean and Middle East region, where progenitors of many cultivated crops occur and have to cope with water availability during the vegetation period.

CWRs provide a key to counteract the effects of climate change on the world’s food supply. To fully understand and exploit this process, studies in the geographical centers of origin are needed that combine ecology, physiology and genetics. With modern genomics tools, geospatial analysis combined with systematic phenotyping, it is highly desirable to revisit wild accessions and prioritize their use for breeding tolerance to various stresses. In particular, some local adaptations found in CWRs might maximize fitness in specific habitats, especially in the current climate change context (Warschefsky et al. 2014).

Environmental conditions such as soil salinity, cold and drought stress represent a major constraint on agricultural productivity. Water availability is a primary factor controlling the distribution of vegetation over the earth’s surface. Crop yields are more dependent on an adequate supply of water than on any other single factor, and environmental stress (cold, drought and salt) represents the primary cause of crop yields losses (Boyer 1982, Varshney et al. 2021a). Many other environmental stress factors, such as cold, salt and high temperature, have a water stress component.

Salt stress is one of the limiting factors to achieve their yield potential. However, some wild relatives are reported to possess great adaptability to saline areas and thus may serve as sources for the genes conferring tolerance to high salinity. For instance, whole genome resequencing and high-density quantitative trait locus (QTL) mapping revealed a novel gene-*GmCHX1*-conferring salt tolerance to wild soybean *G. soja* (Qi et al. 2014). Similarly, wild lentils (*Lens odemensis* and *Lens tomentosus*) were identified as displaying tolerance to drought (Gorim and Vandenberg 2017), and adaptation to drought-prone areas was also evident in wild chickpeas (Toker et al. 2007). Reduced transpiration rates or a deep rooting system enable wild lentils to avoid or tolerate drought stress under moisture-controlled conditions (Gorim and Vandenberg 2017). Variation was found in the ability of lentil plants to maintain growth in water-deficit conditions (Singh et al. 2013), and root mass was positively correlated with the chlorophyll content and shoot mass in response to drought (Idrissi et al. 2015). Similarly, wild groundnut (*Arachis duranensis*) maintains a relatively high rate of transpiration and photosynthesis rate under dehydration treatment (Vinson et al. 2018). Adaptive strategies of CWRs particularly in relation to rainfall gradients existing in the Mediterranean environment were shown in narrow-leaf and yellow lupin (Berger et al. 2008), where the occurrence range and environment gradients have influenced the phenology with an impact on seed size and seedling vigor (Berger et al. 2017). Wide variation in shoot and root responses to water deficit was found in faba bean, including canopy temperature and stomatal conductance (Khan et al. 2007, Khazaei et al. 2013a, Belachew et al. 2018). Recent evaluation of the new *Cicer* collection has identified *C. reticulatum* accessions that can set pods earlier and at lower temperatures than the domesticated crop (Von Wettberg et al. 2018).

Two loci *rhg1* and *Rhg4* have been identified from wild soybean controlling resistance against the soybean cyst nematode (SCN), which is one of the major threats limiting soybean production (Shi et al. 2015, Yuan. et al. 2016). Similarly, wild *Cicer* species show broad spectrum of resistance or tolerance against several biotic stresses including *Fusarium* wilt, leaf miner, bruchids and nematodes (Singh and Ocampo 1997). The rich diversity of the nucleotide-binding site-leucine-rich repeat family of resistance genes in CWRs and landraces paves the way for pyramiding or stacking different genes in a single elite variety (Varshney et al. 2013).

Response of wild *Arachis* species against root-knot nematodes (*Meloidogyne arenaria*) and drought was evident in a recent research (Mota et al. 2021). The genes responsible for the combined stress response are involved in the ethylene biosynthetic pathway, and the candidate gene endochitinase-encoding gene (*AsECHI*) from *Arachis stenosperma* reduced the infection rate by 30% as well as accelerating post-drought recovery. In pigeonpea, CWRs are known to possess resistance against a variety of biotic and abiotic stresses (Khoury et al. 2015), whereas wild common bean can tolerate both drought and sub-zero temperatures (Souter et al. 2017). Wild peas including *Pisum fulvum* have been a promising source for incorporating pest and disease resistance into cultivated pea (Byrne et al. 2008, Aryamanesh et al. 2012, 2014). Legume CWRs are also known to contain genes that could enhance the yield of modern varieties. Conservation and evaluation of germplasm, genomics-assisted breeding and multi-omics approaches combined with machine learning will likely greatly help in overcoming the performance gap between the cultivars and CWRs.

## Domestication Syndrome Traits in Grain Legumes

Domestication syndrome (DS) encompasses features that are absent in the wild progenitors or ‘differentially’ manifested in cultivated species, such as changes in seed retention, seed size, inflorescence and plant architecture and flowering synchronization. Meyer and Purugganan (2013) suggested domestication to be a multistage process accompanied by diversification and improvement. Shattering and seed dormancy have been considered as the two most crucial DS traits (Fuller and Allaby 2018, Parker et al. 2021). In the case of cereals, an increase in grain size occurred prior to loss of shattering (Fuller 2007). Grain size enlargement was evident much earlier in the Poaceae than in Fabaceae, and agricultural technologies including plows (or ards) put the selection pressure for enhancing seed size in grain legumes.

There is an ongoing debate about the number of domestication events and centers for a given crop. Two domestication centers have been reported for common bean: Central America and the Andes (Bitocchi et al. 2017), and potential DS traits for this species include determinacy, photoperiod sensitivity, seed coat anthocyanin, pod string, pod dehiscence, seed dormancy and phaseolin, seed weight and seed size.

Legume CWRs show strong seed dormancy, which was beneficial during early time as this trait favored seed dispersal to diverse geographical areas (Smýkal et al. 2014a, 2018), leading to adaptation and evolution (Abbo et al. 2014). *GmHs1-1*, encoding a calcineurin-like metallophosphoesterase transmembrane protein, was found to influence seed coat permeability in soybean (Sun et al. 2015). An SNP in the genomic region containing *qHS1* coding for endo-1,4-β-glucanase was identified by Jang et al. (2015). The question remains whether this might be a consequence of more than one domestication event in soybean or these genes act in the same pathway.

Recently, insertion resulting in frameshift in the pectin acetylesterase (*PAE 8*) gene was implicated in seed coat permeability in common bean (Soltani et al. 2021). Non-functional *pae 8* allele resulted in an about 2.5-fold increase in the acetylation of soluble pectin in the mature seed coat and consequently increased the rate of water absorption (Palmer et al. 2021). Respective genes underlying seed dormancy in pea, lentil and chickpea remain to be identified yet (Hradilová et al. 2017). In contrast to vernalization-insensitive cultivated chickpea, *C. reticulatum* accessions exhibit considerable flowering advances in response to vernalization. According to Abbo et al. (2014), vernalization is one of the major adaptation traits of wild chickpea native to Southwest Turkey.

### Seed pigmentation and domestication

The seed coat color is mainly determined by polyphenolic substances. Notably, there is a loss of seed pigmentation associated with domestication (Smýkal et al. 2014a, b, Paauw et al. 2019). The dark seed coat color is hypothesized to provide seed protection from foragers (Porter 2013). Indeed, the seed coat color of the wild chickpea progenitor was shown to correspond to soil color (Von Wettberg et al. 2018), and this was removed during chickpea domestication (Varma Penmetsa et al. 2016). The loss of seed pigmentation is associated with the content of alkaloids and other bitter taste compounds (Ku et al. 2020). The change in seed coat color may result from the loss of function of a particular gene. For instance, stay-green type seed coat color in chickpea is the result of dysfunctional chlorophyll degradation pathways and hence extended retention of chlorophyll in all plant organs due to the loss of function of ‘*CaStGR1*’ (Kaliamoorthy et al. 2019). There is an unresolved question if the seed color was selected directly, as a result of cultural preference or indirectly as being associated with seed dormancy and metabolites (Hradilová et al. 2019). The stay-green genotype (G) of wild soybeans has been associated with dormancy, and the trait was lost during soybean domestication, with yellow varieties carrying the mutant allele (Wang et al. 2018). Soybean seed pigmentation is provided by the dominant alleles of the *I* locus that silence the expression of the chalcone synthase genes (Tuteja et al. 2009), leading to reduced biosynthesis of flavonoids. Notably, anthocyanin and flavonol biosynthesis in flowers and seed coats depends on homologous genes encoded by the bHLH transcription factor in several legumes, such as *P* locus in common bean (McClean et al. 2018), Mendel’s *A* locus in pea (Hellens et al. 2010), *B* locus in chickpea (Varma Penmetsa et al. 2016) and, finally, *Tan* gene in lentil (Mirali et al. 2016). However, two homologous genes have been identified in faba bean (Gutierrez and Torres 2019, Gutierrez et al. 2020).

The so-called condensed tannins, involved in seed coat pigmentation, are considered to be anti-nutritional factors that negatively influence digestion, especially in monogastric animals. On the other hand, these aromatic molecules fulfill important biological functions in plant development and defense, including signaling for pollinators and plant protection against fungal pathogens, insect pests and other herbivores.

### Post-domestication diversification and improvement

**Fig. 1:**
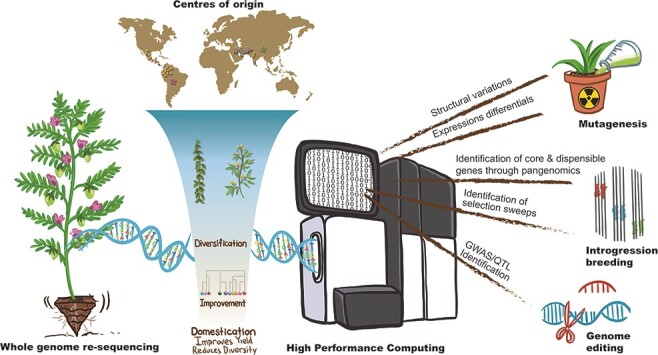
Understanding domestication of grain legumes for future improvement. Grain legumes have been domesticated in different centers of origins worldwide and then spread to diverse eco-geographies for crop production. Morpho-physiological traits during domestication and diversification/improvement show distinct phenotypes among wild and current breeding pools. In recent years, incorporation of omics-level data and pangenome has strengthened crop genetic research. Once the key domestication loci are precisely mapped, the CWRs can be readily converted into domesticated forms by transferring these loci into CWRs through gene editing, introgression and mutagenesis approaches. Rapid domestication of CWRs using advanced biotechnologies will be key to sustainable crop improvement.

To disentangle the crucial DS traits from those involved in post-domestication diversification, Abbo et al. (2014) suggest that the crucial DS traits are mono or di-genic, with domesticated phenotypes conferred by recessive alleles. DS traits such as seed dormancy loss in Near Eastern grain legumes show a marked dimorphism between the wild ancestors and domesticates (Smýkal et al. 2014a, Hradilová et al. 2017, Sedláková et al. 2021). By contrast, the traits showing a phenotypic continuum spread across both wild and cultivated pools are unlikely to be ‘essentially required’ for the survival and propagation of the crops (**Fig. 1**). The free germination of cultigens in pea (Abbo et al. 2013) and lentil (Ladizinsky 1993) endorses this conceptual distinction. Archeological evidence, QTL mapping and recent genome sequencing (Kreplak et al. 2019) further reinforce the fact that free germination is a prominent DS trait in pea (Smýkal et al. 2014a).

Agricultural practices adopted by early farmers exposed crops to selection pressures, and few of these practices drove adaptation after domestication. A notable example includes a transition in the crop growing season of chickpea from autumn to spring due to higher susceptibility to ascochyta blight disease during the rainy season (Ortega et al. 2019). The lack of a vernalization response allowed domesticated chickpea to adapt to this shift (Abbo et al. 2003). In soybean, determinate and semi-determinate growth (Ping et al. 2014), flowering and maturity (Langewisch et al. 2014), seed coat color and hilum color, flower color and pubescence evolved diversification. However, improvement influenced several other traits like shattering, pod color, SCN resistance, leaf shape, four seed per pod and diversity in glycosylation and oil content (Zhou et al. 2015a, b). Semi-determinacy in soybean is caused by a gain-of-function mutation in the gene (*Dt2*), and this gene not only represses *Dt1* that prevents terminal flowering but also activates genes that promote flowering (Zhang et al. 2019).

A comparative analysis of the domestication traits could further enlighten the molecular basis of evolutionary parallelism in crops (Fuller et al. 2014). A gene responsible for seed shattering (*Sh1*) is common and well conserved in legumes and cereals (Lin et al. 2012), and the gene network controlled by *Sh1* tends to converge under artificial selection. Ladizinsky (1979) found that the loss of pod shattering in lentils is regulated by a single locus *Pi*, while in chickpea shattering is oligogenic. Earlier studies in cowpea (*Dhp*) and pea (*Dpo*) demonstrated it to be a monogenic trait; however, later several QTLs affecting this trait were also discovered (Ogutcen et al. 2018). Similarly, in pea, major *Dpo1* loci and minor *Dpo2* were identified (Weeden 2007), and its candidate gene was identified by transcriptomic analysis (Hradilová et al. 2017). Seen in this light, loss of pod shattering is considered a recessive trait evolved during domestication; however, later a higher demand and artificial selection engaged several other loci for adaptability (Ogutcen et al. 2018). More recently, Varshney et al. (2021b) found an allelic variation at *SHATTERPROOF2* homolog on chromosome 5 that is responsible for low or minimum pod shattering in cultivated chickpeas.

Besides seed dehiscence and dormancy, flowering time is the key to successful crop production. Functional homologs of the *Arabidopsis* terminal flowering gene, *TFL1*, associated with determinate growth in grain legumes such as common bean, *PvTFL1y*, and pigeonpea, *CcTFL1Y*, indicate artificial selection pressure for the determinacy trait (Repinski et al. 2012, Mir et al. 2014). Similarly, orthologs of the circadian clock gene *EARLYFLOWERING* (*ELF3*) underlying flowering time are also known to affect flowering in soybean (Yue et al. 2017), chickpea (Ridge et al. 2017), lentil and pea (Weller et al. 2012). Wild forms of *L. angustifolius* require vernalization (cold treatment) in order to promote flowering. In course of narrow-leaf lupine domestication, this requirement has been removed and widely exploited by plant breeders to develop early-flowering varieties of *L. angustifolius*. It has been shown that the loss of vernalization requirement in narrow-leaved lupin is associated with a deletion in the promoter and de-repressed expression of a Flowering Locus T (FT) homolog (Nelson et al. 2017). Similarly, altered expression of genes underlying FT cluster is responsible for domestication-related changes to phenology and growth habit in chickpea (Ortega et al. 2019).

## Understanding Genetic Architecture of Domestication and Improvement Traits

Genetic linkage analysis and QTL mapping have resulted in the identification of major genes controlling domestication traits in several crops including grain legumes. In this section, we discuss different approaches that reveal important regions of the genome potentially involved in shaping the genetic architectures of these traits.

### Classical genetics analysis and QTL mapping

Classical genetics and QTL mapping studies based on bi-parental populations involving wild and domesticated forms have achieved considerable success in the identification and cloning of genomic loci controlling major domestication traits (Doebley et al. 2006). For example, genetic analysis of crosses among the parents representing different stages of the domestication level has provided 20 QTLs for modifications in plant forms in common bean (Weeden 2007). Similarly, wild to cultivated crosses in mungbean and yardlong bean (Isemura et al. 2012, Kongjaimun et al. 2012) revealed QTLs for 38 domestication-related traits or single-nucleotide polymorphisms derived from 670 genes.

In the legumes, single locus control of pod dehiscence was found in lentil (Ladizinsky 1993), pea (Weeden 2007) and chickpea (Ladizinsky 1979), while two loci were found in mungbean (Isemura et al. 2012) and yardlong bean (Kongjaimun et al. 2012). Mapping of the genes controlling pod shattering to a syntenic region in pea and lentil suggested modification of the same genes during the domestication of the two cool-season legumes (Weeden et al. 2002, Weeden 2007). In soybean, the major QTL controlling pod dehiscence is *qPDH1* (QTL for Pod Dehiscence 1), encoding a dirigent-like protein expressed in the sclerenchyma of differentiating endocarp and modulating the mechanical properties of the pod through lignin biosynthesis (Suzuki et al. 2010, Funatsuki et al. 2014). **Table 2** presents a list of the QTL studies in grain legume crops that have reported genetic dissection of domestication traits. Recently, QTL-seq in inter- (*C. arietinum* ICC 4958 × *C reticulatum* ICC 17160) and intra- (ICC 4958 × *C. arietinum* ICC 8261) specific recombinant inbred lines revealed major QTL for flowering time in chickpea (Srivastava et al. 2017). Like other legumes including soybean (Watanabe et al. 2011, Wang et al. 2016), pea (Hecht et al. 2007) and common bean (Bellucci et al. 2014), this study suggested a role for the mutation in the *GI* (GIGANTEA) ortholog during chickpea domestication through conferring flowering time adaptation.

**Table 2:** Identification of genomic loci and candidate gene associated with the process of domestication, diversification and improvement

Crop	Trait	Category	Gene	Reference
Soybean	Shattering	Domestication	*GmSHAT1-5*	Dong et al. (2014)
	Hard seededness	Domestication	*GmHs1-1*	Sun et al. (2015)
	Determinate growth	Diversification	*GmDt1*/*GmTFL1b*	Liu et al. (2010), Tian et al. (2010), Zhou et al. (2015a)
	Semi-determinate growth	Diversification	*GmDt2*	Ping et al. (2014)
	Flowering time	Diversification	*GmCRY1a*	Zhang et al. (2008),
	Flowering time	Diversification	*GmTFL1a*	Li et al. (2013)
	Flowering time	Diversification	*GmCOL7a*	Li et al. (2013)
	Flowering and maturity	Diversification/improvement	*E1; E2* (*GmGIa*); *E3* (*GmPhyA3*); *E4* (*GmPhyA2*)	Watanabe et al. (2011), Langewisch et al. (2014)
	Seed coat color, hilum color	Diversification	*I* (*GmCHS*)	Tuteja et al. (2009), Zhou et al. (2015a)
	Flower color	Diversification	*W1* (*F3*′*5*′*H*)	Zhou et al. (2015a)
	Pubescence	Diversification	*T*	Zhou et al. (2015a)
	Cyst nematode resistance	Improvement	*Rhg1*	Cook et al. (2012), Zhou et al. (2015a)
	Pod color	Improvement	*L1* (*MYB*)	He et al. (2015)
	Shattering	Improvement	*GmPdh1*	Funatsuki et al. (2014)
	Leaf shape and four seed pod	Improvement	*Ln*	Zhou et al. (2015a)
	Oil content	Improvement	*LPD1*	Qi et al. (2011), Zhou et al. (2015a)
	Structural diversity of glycosylation	Improvement	*Sg-1*	Zhou et al. (2015a)
	Flowering and maturity	Improvement	*Tof12*	Lu et al. (2020)
	Salt tolerance	Improvement	*GmCHX1*	Qi et al. (2014)
Common bean	Pod dehiscence	Domestication	*PD; PvIND*	Gioia et al. (2013), Parker et al. (2020)
	Resistance and tolerance gene	Improvement	*AN-Pv33, AN-Pv69, AN-DNAJ* and *Leg223*	Bitocchi et al. (2017)
	Photoperiodic response	Improvement	*GIGANTEA* (*GI*)	Bellucci et al. (2014)
	Vernalization	Diversification (Mesoamerican)	*VRN1* (*Phvul.003G033400*) and *VRN2* (*Phvul.002G000500*)	Schmutz et al. (2014)
		Diversification (Andean)	*FRL1* (*Phvul.006G053200*) and *TFL2* (*Phvul.009G117500*)	Schmutz et al. (2014)
	Flowering	Diversification (Mesoamerican)	*COP1*	Schmutz et al. (2014)
		Diversification (Andean)	*CUL4* along with *COP1*	Schmutz et al. (2014)
			*FUL* and *AGL42*	Schmutz et al. (2014)
	Nitrogen metabolism (nitrate reductase)		*Phvul.008G168000; Phvul.005 G132200*	Schmutz et al. (2014)
	Nitrogen transporter		*Phvul.002G242900*	Schmutz et al. (2014)
Chickpea	Vernalization	Diversification	*VRN1*	Varshney et al. (2019)
	Shattering	Domestication	*Shatterproof2*	Varshney et al. (2021b)
	Flower development and reproductive phase transition of meristem	Diversification	*FLP2*	Varshney et al. (2021b)
	Flowering time	Diversification	*DTF3A*	Ortega et al. 2019
	Root growth		*LRP1*	Varshney et al. (2021b)
	Signaling pathways for survival and T cell metabolism		*PIP5KL1*	Varshney et al. (2021b)
	Flavonoid biosynthesis		*MYB12*	Varshney et al. (2021b)
Groundnut	Seed size/weight	Domestication	*TGA7, topless-related protein 2, IAA-amino acid hydrolase ILR1-like 5* and *putative pentatricopeptide repeat-containing* (*PPR*) *protein*	Li et al. (2021)
Lentil	Pod indehiscence	Domestication	*Pi*	Sonnante et al. (2009)
	Growth habitat		*Gh*	Sonnante et al. (2009)
	Cotyledon color	Improvement	*Yc*	Sonnante et al. (2009)
	Epicotyl color	Improvement	*Gs*	Sonnante et al. (2009)
	Seed coat spotting	Improvement	*Scp*	Sonnante et al. (2009)
	Seed ground color	Improvement	*Ggc*	Sonnante et al. (2009)
	Flower color	Improvement	*W*	Sonnante et al. (2009)
Pea	Dehiscent pod	Domestication	*Dpo*	Blixt (1972), Hradilová et al. (2017)
Pigeonpea	Shattering	Domestication	*SHATTERING1*(*C.cajan_24676*)	Varshney et al. (2017), Ogutcen et al. (2018)
	Flowering	Improvement	*EARLY FLOWERING3* (*ELF3*)(*C.cajan_22378*), *FLOWERING LOCUS C A10*(*C.cajan_18407*), *TERMINAL FLOWER1b*(*C.cajan_10074*), *FLOWERING TIME1*(*C.cajan_42877*)	Varshney et al. (2017)
	Transparent testa	Improvement	*C.cajan_01740*	Varshney et al. (2017)
	Seed-related features	Improvement	*Granule bound starch synthase I* (*C.cajan_02047*), *AcruGRANULE BOUND STARCH SYNTHASE I*(*C.cajan_07051*), *FRUIT WEIGHT*(*C.cajan_15593*), *SUGARY1*(*C.cajan_2320*)	Varshney et al. (2017)
	Semi-dwarf	Improvement	*C.cajan_04115*	Varshney et al. (2017)
	Determinacy	Diversification	*CcTFL1*	Mir et al. (2014)

The exotic genetic libraries/introgression libraries generated by backcrossing an elite parent with a landrace or CWR accession offer valuable resources to find the genomic loci associated with domestication traits (see Bohra et al. 2022). For example, introgression lines or chromosome segment substitution lines (CSSLs) developed from wild and domesticated accessions allowed the detection of the wild donor haplotypes or genetic segments associated with domestication traits in soybean (Wang et al. 2013, He et al. 2015, Yang et al. 2017, 2019, Liu et al. 2021), for agronomic and seed size-related traits (Blair et al. 2006), and pod dehiscence (Rau et al. 2019) in common bean and oil content, days to maturity, plant height and seed composition in groundnut (Fonceka et al. 2012). The presence of favorable alleles of QTL in wild species uncovered their potential for improvement of legume crops (Blair et al. 2006). The changes in morphological and physiological traits that distinguish wild and cultivated forms could be largely attributable to major QTL; however, detection of minor QTLs such as for seed size in soybean (Liu et al. 2007) suggests domestication as a slow evolutionary change resulting from the accumulation of minor changes in the QTL.

### Genome-wide association studies

A genome-wide association study (GWAS) is an alternative approach to associate genomic variations with domestication phenotypes in diverse germplasm collection. GWAS of 203 accessions combined with transgenic and RNA-Seq experiments in groundnut facilitated the identification and validation of genes for seed weight (*AhFAX1*) and seed length (*AhDPB2*) (Liu et al. 2022). Likewise, GWAS of domestication traits identified 29 SNPs and candidate genes for seed traits in soybean (Leamy et al. 2017) and a total of 262 gene-trait associations and candidate genes for drought and heat stress tolerance in chickpea (Varshney et al. 2019). By using wild pea diversity (*P. fulvum, Pisum hilum, Pisum elatius, Pisum speciosum, Pisum transcaucasium* and *Pisum abyssinicum*), 62 SNPs showed association with frost tolerance and provided superior haplotypes associated with the frost damage (Beji et al. 2020). The contributions of large SVs toward shaping domestication and improvement traits have been evident following resequencing of multiple accessions and GWAS, as exemplified by the detection of CNVs for plant height, oil content, seed weight, seed coat color (Zhou et al. 2015b) and PAV for seed luster (Liu et al. 2020) in soybean. Genome-wide analysis provides new insights into diversity, distribution and hybridization of the wild species. In pea, Smýkal et al. (2017) suggested that Miocene–Pliocene events shaped the genetic diversity of wild pea, and Pleistocene–Holocene climatic changes were responsible for its phylogenetic diversity. Recently, RAD-sequencing of 494 pea samples confirmed two genetically independent domestication events in pea (Hellwig et al. 2022) corresponding with earlier reports (Trněný et al. 2018). Other GWA analyses performed recently in grain legumes have elucidated the genetic architectures of the domestication and improvement traits such as morpho-agronomic traits in groundnut (Pandey et al. 2014) and common bean (Delfini et al. 2021) and flowering time variation (Raggi et al. 2019), pod indehiscence (Parker et al. 2020), pod morphological and color characters (García-Fernández et al. 2021) and seed weight (Schmutz et al. 2014) in common bean. The high density of markers has allowed genome-wide surveys of selection signals. In pigeonpea, the GWAS revealed an abundance of gene-trait associations on CcLG09 (Varshney et al. 2017). The study suggested that extensive LD blocks resulting from genetic hitchhiking implied a strong impact on this particular chromosome during domestication and improvement. A better understanding of the genetic basis of domestication phenotypes in combination with the innovative breeding technologies will help introduce the useful characters from the wild into the cultivated genetic background.

### Multi-omics approaches

Recent expansion in our capacities to incorporate omics-level data into genetic research has helped comprehend the process of crop domestication. These studies suggest a common trend of reducing allelic diversity (Smýkal et al. 2018), and it is also claimed that our current major crops contain only 6% of the allelic diversity present in wild gene pools (Fernie et al. 2006). Seed coat color is determined by tannins, and the loss of pigment in the seed coat of cultivated beans presents an obvious example of the effects of domestication (McClean et al. 2018). Potential alterations have been reported in the production of secondary metabolites during domestication (Ku et al. 2020). Transcriptome analysis of the leaf development stages reported a nearly 60% reduction in nucleotide diversity and an 18% reduction in gene expression diversity in the domesticated forms of common bean as compared with the wild reduction (Bellucci et al. 2014). Half of the contigs reached fixation following domestication, and the study supports the role of both directional and diversifying selection in Mesoamerican common bean. In chickpea, a major QTL containing 59 genes was mapped on chromosome 3 and found to exhibit pleiotropic effects on flowering time and growth habit. The higher expression of this gene cluster in domesticated chickpea as compared to wild *C. reticulatum* supported its contribution to early phenology, crucial to transitioning from winter to summer crop (Ortega et al. 2019). Domestication has influenced the synthesis of primary as well as secondary metabolites. Association of phenotype variations has been reported with specific metabolic traits such as oil content, aroma, sweetness and antioxidants contents. Integration of next-generation sequencing with metabolomics has facilitated comprehensive analysis of the metabolites playing roles in biological processes and pathways associated with crop domestication (Zhu et al. 2014). For instance, nuclear magnetic resonance analysis in soybean revealed variability in the primary and secondary metabolites in seeds and leaves of a cultivar, a landrace and wild *G. Soja* (Yun et al. 2020). The authors proposed metabolic variations in the acquisition of adaptations to different environments. In pea, investigation of two important domestication traits, i.e. pod dehiscence and seed dormancy, by integrating transcriptomic, metabolomic and anatomic data revealed considerable differences in the texture of testa surfaces, length of macrosclereids and seed coat thickness (Hradilová et al. 2017). Application of multi-omics data to genetic studies not only helps narrowing down to the causative gene(s) but also becomes extremely important considering the safety aspects of new food crops developed through genetic engineering or gene editing.

### Pangenomics

A pangenome is composed of the entire set of genes within a species, and the concept is now extended to the genus level (Khan et al. 2020). In a pangenome, core genes are shared across all individuals, whereas the remaining genes belong to the dispensable genome (Khan et al. 2020). In legumes, the first pangenomic study was conducted in soybean (Li et al. 2014), followed by pigeonpea (Zhao et al. 2020) and chickpea (Varshney et al. 2021b). The analysis of soybean pangenome based on seven *G. soja* accessions and a cultivated type (*G. max*) led authors to report a significant loss of diversity during domestication, and most of the diversity (87%) was found in the wild (Li et al. 2014). The pangenomic analysis also paved the way for the identification of SVs in the genic regions; of 1,978 genes affected in wild accessions, 1,179 had CNV loss, 726 had CNV gain and 73 had both loss and gain in wild as compared to cultivated. The gene model used suggested a comparatively higher number of genes in *G. soja* (55,570) than *G. max* (54,175). Decades of research have enlightened the impact of SVs (deletion, insertion, inversion, duplication and chromosomal rearrangements) in crop evolution and agricultural adaptations. Compared to SNPs, SVs can directly alter the genic expression by modifying the copy number and can cause large-scale perturbations of *cis*-regulatory regions (Lye and Purugganan 2019). In another study, de novo genome assemblies of 26 representative soybeans selected from 2,898 deep-sequenced accessions identified a total of 723,862 PAVs, 27,531 CNVs, 21,886 translocation events and 3,120 inversion events (Liu et al. 2020). This study reported a total of 28,786 genes as core genes and 28,706 as dispensable genes. Congruent with previous findings, this study reinforced that one obvious trait that was selected during soybean domestication is seed coat color, with wild exhibiting black seed coat color, while domesticates show yellow coat color. This seed coat color is found to be associated with reduced chalcone synthase (*CHS*) gene expression in yellow soybean (Tuteja et al. 2004) seed coats via homology-dependent gene silencing, which is caused by SV of inversion and gene duplication of the CHS cluster arising from double crossover events (Xie et al. 2019). Concerning PAVs potentially associated with soybean domestication, a 360-kb inversion was observed on chromosome 7 distinguishing cultivated and wild soybean, and the event could have occurred nearly 4,700 years ago (Liu et al. 2020). The deletion and duplication can be considered of high importance in both plants and animals as these alter the copy number and expression of dosage-sensitive genes to modify phenotypic diversity, including traits important in domestication and breeding. Although not performed in legumes, research on other major crops such as tomato highlights the impact of dosage sensitivity in genes by gene-editing methodologies (Alonge et al. 2020, Alseekh et al. 2020, Domínguez et al. 2020). In pigeonpea, Zhao et al. (2020) developed a pangenome with 48,067 core (86.6%) and 7,445 dispensable genes (13.4%). A recent chickpea pangenome with 29,870 gene models sheds light on various aspects such as domestication, species divergence and adaptation and provides superior haplotypes for agronomic traits (Varshney et al. 2021b). A total of 793 genes gained CNVs and 209 genes lost CNVs in the cultivated accessions, whereas wild chickpea had 643 and 247 genes showing gain and loss of CNVs, respectively. Growing numbers of high-quality de novo genome assemblies along with refined methods of pangenome development will provide crop researchers with better tools and opportunities to recapture the genetic diversity that has been lost during crop domestication and evolution.

## Incorporating CWRs for Grain Legume Improvement in the Genomics Era

Knowledge of the genetic architectures of domestication and adaptation traits enables researchers to make the cultivated crops enriched with traits that enhance crop production in current agricultural settings. In this section, we highlight three key approaches to accelerate the transfer of beneficial traits from wild to domesticates.

### Genomics-assisted approaches

Large-scale genome sequencing efforts in grain legumes have opened enormous opportunities to harness beneficial genetic variations archived in the global genebanks (McCouch et al. 2013). Alternatively, these germplasm sets can be customized to a manageable scale for efficient use in breeding programs in the form of core and min-core collections. Focused identification of the germplasm strategy, an eco-geographic approach, has been extensively applied in grain legume crops such as faba bean (Khazaei et al. 2013b) and soybean (Haupt and Schmid 2020) for the identification of promising germplasm containing traits important for adaptation. Focused identification of the germplasms and an appropriate breeding strategy are urgently needed to introduce new genes into the elite pool. We have recently reviewed approaches to identify and incorporate beneficial genetic variations from CWRs and landraces (Bohra et al. 2022). One of such approaches is the advanced backcross-QTL based on cultivated Andean bean Cerinza and wild G24404 detected QTL for phenology (Blair et al. 2006) and mineral traits (Blair and Izquierdo 2012). The presence of favorable alleles in wild bean for 13 QTLs controlling plant height, yield and yield components and seed size showed the potential of AB-QTL for uncovering new genetic variations for bean improvement. Similarly, AB-QTL in pigeonpea based on two backcross populations ICPL 87119 × ICPW 15613 and ICPL 87119 × ICPW 29 led to the identification of a total of 86 QTL (12–21% phenotypic variation explained) and 107 QTL (11–29% PVE), respectively (Saxena et al. 2019). A similar AB-QTL analysis in groundnut based on populations ICGV 91114 × ISATGR 1212 and ICGV 87846 × ISATGR 265-5A demonstrated favorable alleles in genomic segments from wild groundnut for improving resistance against foliar diseases. The synthetic allotetraploid (AABB) ISATGR 1212 and autotetraploid (AAAA) ISATGR 265-5A were developed from the crosses *A. duranensis* ICG 8123 × *Arachis ipaensis* ICG 8206) and *Arachis kempff-mercadoi* ICG 8164 × *Arachis hoehnei* ICG 8190), respectively (Khera et al. 2019). As demonstrated in pea, cross combinations involving CWRs, such as *P. fulvum*, can create novel transgressive segregants for agro-morphological traits. A super early progeny recovered in an interspecific population flowered 13–17 d and had pod setting in 18–29 d after germination (Sari et al. 2021). The study also revealed that this progeny can be heat tolerant due to its escape ability. The breeding cycle can be further accelerated using in vitro embryo culture or speed breeding techniques. Another approach is the construction of CSSL libraries that contain overlapping genomic segments from the CWRs or landraces. Construction of CSSL libraries in soybean facilitated the selection of superior haplotypes for agronomic traits such as days to flowering and seed coat color (Liu et al. 2021). The groundnut CSSL library was created using the wild synthetic allotetraploid (*A. ipaensis* × *A. duranensis*), and the QTL analysis identified 42 potential QTL governing significant PVs for plant growth habit, height, plant spread and flower color (Fonceka et al. 2012). Similar CSSLs containing genomic segments of wild pea (*P. fulvum* or *P. elatius*) in the genetic background of *P. sativum* offer a valuable resource for genetic research and breeding (Zablatzká and Smýkal 2015). This method is particularly suitable for prebreeding and germplasm development programs. The reader may refer to Bohra et al. (2022) for details on the breeding strategies to improve plant traits using CWRs. The new breeding techniques supported by informed germplasm collection strategies would be crucial to enhance breeder’s access to valuable CWRs and their rapid deployment in grain legume improvement.

### De novo domestication

De novo domestication refers to introducing the domestication genes back into CWRs or lesser-grown species using classical, mutagenesis or gene-editing approaches (Fernie et al. 2021, Zsögön et al. 2022, **Fig. 1**). Given that the majority of the domestication-related loci reported to date represent loss-of-function mutants, intentional modification of these loci in CWRs is easier than genetic manipulation of adaptation-related architectures in modern cultivars (Zsögön et al. 2018, Takahashi et al. 2019). Since the time of domestication, the humans/breeders made selections in favor of traits rendering cultivated crops easier to breed, culture and store seeds and tried to stack them, leading to the pyramiding of valuable mutations and recombinants at key genomic loci. During the process, however, domestication involved a cost in the form of accumulated genetic load while shaping the genetic architectures of current phenotypes of desirable traits. In this context, the targeted nature of de novo domestication provides an efficient way to address deleterious mutants that have accompanied selected loci during domestication. Pioneering experiments on de novo domestication using gene editing have shown rapid improvement of domestication traits in Solanaceae crops (Fernie and Yan 2019, Bohra et al. 2022). Three studies demonstrated improvement in domestication traits of wild tomato (*Solanum pimpinellifolium*) by using clustered regularly interspaced short palindromic repeats (CRISPR)/CRISPR-associated protein 9 (Cas9) editing of key domestication loci such as *fas, lc, CLV3* (Rodriguez-Leal et al. 2017), *SP, SP5G, SlCLV3, SlWUS, SlGGP1* (Li et al. 2018) and *SP, O, FW 2.2* and *CycB* (Zsögön et al. 2018). Two studies in ground cherry (*Physalis pruinosa*), an orphan Solanaceae crop, showed the potential of CRISPR–Cas9 editing for de novo domestication by altering one (*SlER*; Kwon et al. 2020) and three loci (SP, SP5G and CLV; Lemmon et al. 2018). More recently, rapid improvement of the six agronomic traits of allotetraploid rice (*Oryza alta*) was achieved by Yu et al. (2021) by establishing a high-quality genome assembly and efficient transformation system that in turn paved the way for CRISPR/Cas9 genome editing of the allotetraploid rice. Other successful examples of de novo domestication have been published in different crops including rice, pennycress, sunflower and the legume *Vigna stipulacea.*Takahashi et al. (2019) combined ethyl methanesulfonate (EMS) mutagenesis with the forward phenotypic screening to enable de novo domestication of *V. stipulacea*, resulting in the recovery of mutants with notable reductions in seed dormancy and pod shattering. Given the species-rich legume family, there is large potential to domesticate novel legume crops for both human food and animal feed. This effort has been recently demonstrated in hairy vetch (*Vicia villosa*) (Kissing Kucek et al. 2020). Another promising approach is the domestication of perennial grain crops. Since traditional systems relying on annual crops have substantial negative impacts on ecosystem functions (e.g. nutrient cycling, water quality and carbon emissions, salinity, soil erosion and degradation) (Pimentel et al. 2012), these problems could potentially be reduced through the introduction of perennial crops, which have more extensive root systems (Schlautman et al. 2018).

While legume CWRs have not yet been subjected to de novo domestication, several traits that could be targeted in these species spring to mind. Perhaps the first of these is seed shattering, which in e.g. common bean (Di Vittori et al. 2021, Parker et al. 2021) as in cereals (Avni et al. 2017, Lv et al. 2018) is an important domestication trait. That said a range of other domestication traits from legumes such as early flowering, determinacy, response to biotic and abiotic stresses and nutritional traits (protein, vitamins, phytonutrients, lack of antinutrients and oil quality) would also represent important targets for de novo domestication. The recent sequencing approaches carried out in a wide range of leguminous species including chickpea (Varshney et al. 2019, 2021b), pigeonpea (Varshney et al. 2017) and groundnut (Chen et al. 2016, Zhuang et al. 2019) are additionally likely to be useful catalogs, in which the past changes that occurred over millennia of domestication can be probed as a source of important key genes for the de novo domestication of underutilized legumes, which represent particularly important potential crops given they represent low-input agriculture.

### Redomestication

The process of redomestication represents an important opportunity for cultivated species to adapt to new ecotype. Besides de novo domestication that could deliver entirely new crops, the wild species or existing crops can be redomesticated by changing the artificial selection pressures toward plant traits catering to future requirements (Tian et al. 2021). At the Land Institute (Salina, Kansas, UK), researchers have targeted some of the perennial legumes for domestication and evaluated potential candidate species (Schlautman et al. 2018). Several species of perennial legumes are not yet fully domesticated, and the idea of using the latest technologies in nucleotide sequencing and precise phenotyping can fast-track the procedure. Genomic loci that might have undergone selection pressures in the past or have the potential to adapt are most suitable candidates for redomestication. For example, the domesticated soybean accessions have a higher oil content than wild seeds (Zhou et al. 2015b). Targeted domestication of new crops according to human needs will bolster global efforts to meet food security targets of the growing population. In the case of legumes, the protein content is also of high importance, particularly given the high interest in moving away from animal-based diets, while legumes are commonly regarded as a great source of protein; this remains highly variable and would be a great target for de novo and redomestication alike.

## Concluding Remarks

Since the beginning of agriculture, grain legumes have been important ingredients as protein complement sources to starch-rich cereal diets. The domestication of cereal crops has been intensively researched, resulting in the identification and cloning of domestication genes. In recent years, carbonized remains and genetic studies have helped establishing the role of grain crops in old as well as new world agriculture. A very recent example of this is the identification of a WD40 gene as being important in the domestication of grain yield in both rice and maize with a wide range of other genes putatively also following this trend (Chen et al. 2022). As such deep and broad sequencing is proving a highly rich source of lead genes for crop improvement—as alluded to in the title of this article, we really are studying our past to inform our future.

Domestication of cereals and grain legumes has shown different trajectories as exemplified from the time lines of grain size enlargement (Fuller 2007, Abbo et al. 2014). Genetic studies have associated crop domestication with a limited number of loci having a strong impact on the domestication phenotypes (**Fig. 1**). Notwithstanding the challenges associated with legume transformation and regeneration, intentional manipulation of major domestication loci using gene editing is a promising approach to create fundamental allelic shifts. Alternative hypothesis suggests distinct genetic architectures of domestication and diversification traits. Genome variation scans elucidated the involvement of several loci scattered throughout the genome. Harnessing the potential of gene-editing approaches for de novo domestication will demand proportionate innovations in agronomy, breeding and cultural practices. A careful examination of potential crops is imperative to de novo domestication and may help to mitigate against the effect of climate change and the associated deterioration in the quality of arable land (Yu and Li 2022, Zsögön et al. 2022).

Indeed, we are convinced that combined evidence from archeobotanical, pangenomes, multi-omics science and cultural shifts will enlighten future crop improvement strategies to rapidly develop modern cultivars of grain legume crops. That said, this will be no easy task and will certainly require both multi-disciplinary and multi-national approaches; however, in principle, the necessary technologies are all in hand and studies concerning the adaptation of our major crops to novel environments will undoubtedly help in efforts to create a more sustainable global agriculture that is more resistant to extreme climacteric events.

## Supplementary Material

pcac086_Supp

## Data Availability

No new datasets were generated or analyzed in this study.
