# Supplementary material for: The Key to the Future Lies in the Past: Insights from Grain Legume Domestication and Improvement Should Inform Future Breeding Strategies
Source: Plant Cell Physiol. 2022 Jun 17;63(11):1554–72. doi: 10.1093/pcp/pcac086 (PMC9680861; doi:10.1093/pcp/pcac086)
Supplement: pcac086_Supp [file pcac086_supp.zip › pcp-2022-e-00085-File005.docx]

**Supplementary Table 1**. Wild grain legumes as source of yield and stress response

| **Crop** | **Trait** | **Donor** | **References** |
| --- | --- | --- | --- |
| Soybean | Salt tolerance | *G.soja* | Qi et al. (2014), Zhang et al. (2019), |
|  | Drought tolerance | *G.soja* | Wang et al. (2019) |
|  | Soybean cyst nematode resistance | *G.soja* | Yuan et al. (2016) |
|  | Foxglove aphid resistance | *G.soja* | Lee et al. (2015), Koh et al. (2020) |
|  | Soybean aphid resistance | *G.soja* | Zhang et al. (2017a,b) |
|  | Common cutworm | *G.soja* | Oki et al. (2019) |
|  | Root architecture (drought adaptations) | *G.soja* | Prince et al. (2015) |
|  | Seed protein coat | *G.soja* | Diers et al. (1992) |
|  | Seed saturated fatty acid content | *G.soja* | Leamy et al. (2017) |
|  | Yield | *G.soja* | Concibido et al. (2003) |
| Common Bean | Anthracnose resistance | *P. coccineus, P. acutifolius, P. lunatus, P. dumosus* | Mahuku et al. (2002), Kumar et al. (2021) |
|  | Common blight resistance (CBB) | *P. acutifolius* | Singh et al. (2001) |
|  | Angular leaf spot resistance | *P. dumosus, P. coccineus* | Mahuku et al. (2003), Nay et al. (2019) |
|  | Cold tolerance | *P. angustissimus, P*. *acutifolius* | Singh et al. (2001) , Cortés et al. (2013) |
|  | Drought tolerance | *P*. *acutifolius* | Souter et al. (2017) |
|  | Salt tolerance | *P. filiformis* | Bayuelo-Jimenez et al. (2002) |
|  | Heat tolerance | *P*. *acutifolius* | Cruz-Ruiz et al. (2021) |
| Chickpea | Ascochyta blight resistance | *C. arietinum, C. bijugum, C.cuneatum, C. echinospermum, C. judaicum, C. montbretii, C.pinnatififidum, C. reticulatum* | Pande et al. (2010), Kaur et al. (2012), Singh et al. (2014), Liet al. (2017) |
|  | Botrytis gray mold resistance | *C. bijugum, C. judaicum, C. pinnatififidum, C. reticulatum* | Pande et al. (2006), Ramgopal et al. (2013), Singh et al. (2014) |
|  | Bruchids resistance | *C. echinospermum* | Eker et al. (2018) |
|  | Fusarium wilt resistance | *C. bijugum, C. cuneatum, C.echinospermum, C. judaicum, C. pinnatififidum, C. reticulatum* | Infantino et al. (1996), Winter et al. (2000), Singh et al. (2005) |
|  | Helicoverpa pod borer resistance | *C. bijugum, C. cuneatum, C.chrossanicum,C. microphyllum, C. canariens,C. macracanthum,C. judaicum* | Sharma et al. (2006), Golla et al. (2018) |
|  | Phytophthora root rot resistance | *C. bijugum, C. echinospermum, C. pinnatififidum, C. reticulatum* | Knights et al. (2008) |
|  | Root knot nematode resistance | *C. bijugum, C. echinospermum, C. judaicum, C. pinnatififidum, C. reticulatum* | Singh et al. (2014) |
|  | Root lesion nematode resistance | *C. echinospermum, C. reticulatum* | Thompson et al. (2011) |
|  | Seed beetle | *C. cuneatum, C. echinospermum, C. judaicum, C. reticulatum* | Gupta and Parihar (2015) |
|  | High number of seeds per plant | *C. cuneatum, C. montbretii* | Singh et al. (2005,2014) |
|  | Cold tolerance | *C. bijugum, C. echinospermum,C. judaicum, C. pinnatififidum, C. reticulatum,C. turcicum* | Saeed and Darvishzadeh (2017), Rani et al. (2020),Toker et al. (2021) |
|  | Heat and drought tolerance | *C. anatolicum, C.echinospermum, C.microphyllum, C. montbretii, C. oxyodon, C. pinnatififidum, C. reticulatum, C. songaricum, Cicer yamashita, Cicer cuneatum* | Canci and Toker (2009a,b), Maqbool et al.(2017), Sharma et al. (2017) |
| Groundnut | Early leaf spot | *A. batizocoi,A. cardenasii, A. duranensis,A. diogoi* | Denwar et al. (2021) |
|  | Rosette disease | *A. villosa,A. stenosperma,A. kuhlmannii;A. hoehnei,A. diogoi,A. cardenasii;A. decora* | Subrahmanyam et al. (2001) |
|  | Bud necrosis virus | *A. benensis,A. cardenasii,A. villosa* | Reddy et al. (1990) |
|  | Rust resistance | *A. valida, A. villosa; A. kuhlmannii; A. ipaënsis* | Subrahmanyam et al.(1983), Pande and Rao (2001) |
|  | Drought tolerance | *Arachis duranensis,A*. *batizocoi* | Dutra et al. (2018) |
|  | Salt tolerance | *Arachis duranensis, A. ipaënsis* | Guimarães et al. (2012), Brasileiro et al. (2015) |
| Lentil | *Ascochyta* blight | *Lens orientalis, L. odemensis, L. nigricans, L.ervoides,L. lamottei* | Ye et al. (2002), Tullu et al. (2010), Rodda et al. (2017), Coyne et al.(2020) |
|  | Stemphylium blight | *Lens tomentosus, Lens orientalis,* L. ervoides or L. lamottei, | Podder et al. (2013), Coyne et al.(2020) |
|  | Fusarium wilt | *Lens tomentosus Lens odomensis, L. orientalis, L.ervoides, L.nigricans, Lens lamottei* | Singh et al. (2020) |
|  | Sitona weevil | *Lens lamottei* | Coyne et al.(2020) |
|  | Powdery mildew | *Lens odomensis, L. orientalis,L.tomentosus, L.ervoides,L.nigricans,L.lamottei* | Singh et al. (2020) |
|  | Rust | *L. orientalis, L.odemensis, L.tomentosus, L.ervoides,L.nigricans* | Singh et al. (2020) |
|  | High pods per plant,shorter internode | *L. orientalis, L.odemensis, L.ervoides* | Singh et al. (2020) |
|  | Cold and drought tolerance | *L. ervoides*, *L. nigricans*, and *L. culinaris* ssp. *orientalis* | Asghar et al. (2021) |
| Pea | Powdery mildew | *Pisum abyssinicum,P.fulvum* | Smýkal et al. (2012), Fondevilla and Rubiales (2012), Bobkov et al. (2021) |
|  | Bruchid resistance | *Pisum fulvum* | Byrne et al. (2008), Aznar-Fernandez & Rubiales (2019) |
|  | Root rot disease | *Pisum fulvum* | Aryamanesh et al. (2012) |
| Pigeonpea | Cytoplasmic male Sterility (CMS) | *C. scarabaeoides,C. sericeus,C. cajanifolius,C. acutifolius* | Dalvi et al. (2008) , Bohra et al. (2010) |
|  | Sterility Mosaic Disease (SMD) | *C. acutifolius, C. albicans, C. crassus, C. lineatus, C. platycarpus,C. scarabaeoides,C. sericeus* | Kulkarni et al. (2003), Kumar et al. (2005) |
|  | *Phytophthora* blight resistance | *C. platycarpus, C. sericeus* | Mallikarjuna et al. (2005), Khoury et al. (2015) |
|  | *Pod borer resistance* | *C. Sericeus,C. Scarabaeoides, C. platycarpus, C. albicans, C. acutifolius* | Sharma et al. (2009) |
|  | Early flowering | *C. scarabaeoides;C. platycarpus* | Das et al. (2020) |
|  | Cleistogamous | *C. lineatus* | Saxena et al. (1992) |
|  | High seed protein | *C. scarabaeoides; C. sericeus; C. albicans* | Sharma and Upadhyaya et al. (2016) |
|  | Dwarfism | *C.scarabaeoides* | Reddy et al. (1990) |
|  | Salt tolerance | *C. platycarpus* | Subbarao (1988), Khoury et al. (2015) |
|  | Drought tolerance | *C.scarabaeoides;C.platycarpus;C. cajanifolius* | Sinha et al. (2020) |
|  | Aluminium toxicity | *C. scarabaeoides;C. platycarpus* | Choudhary et al. (2011) |
|  | Heat stress tolerance | *C. scarabaeoides*; *C. cajanifoliu;* *C. acutifolius*. | Ramakrishna et al. (2021) |

**REFERENCES**

Aryamanesh, N., Byrne, O., Hardie, D.C., Khan, T., Siddique, K.H.M. and Yan, G. (2012) Large-scale density-based screening for pea weevil resistance in advanced backcross lines derived from cultivated field pea (*Pisum sativum*) and *Pisum fulvum*. *Crop Pasture Sci.* 63: 612-618.

Asghar, M. J., Hameed, A., Rizwan, M., Shahid, M. and Atif, R. M. (2021) Lentil Wild Genetic Resource: A Potential source of genetic improvement for biotic and abiotic stress tolerance.In *Wild Germplasm for Genetic Improvement in Crop Plants* (pp. 321-341).Academic Press.

Aznar-Fernández, T., and Rubiales, D. (2019) Flower and pod source influence on pea weevil (*Bruchuspisorum*) oviposition capacity and preference. *Front. Plant Sci.:* 491.

Bayuelo-Jiménez, J.S., Craig,R., and Lynch, J.P. (2002) Salinity tolerance of *Phaseolus* species during germination and early seedling growth. *Crop Sci.* *42*: 1584-1594.

Bobkov, S.V., and Selikhova, T.N. (2021) Introgession of powdery mildew resistance into cultural pea from wild accession of *P. fulvum*. In *IOP Conf. Ser. Earth Environ. Sci.*650 (1): p. 012091.

Bohra, A., Mallikarjuna, N., Saxena, K.B., Upadhyaya, H.D., Vales, I. and Varshney, R.K. (2010) Harnessing the potential of crop wild relatives through genomics tools for pigeonpea improvement. *J. Plant Biol.* 37: 83–98.

Brasileiro, A., Morgante, C.V., Araujo, A.C., Leal-Bertioli, S., Silva, A.K., Martins, A.C.,et al. (2015) Transcriptome profiling of wild Arachis from water-limited environments uncovers drought tolerance candidate genes. *Plant Mol. Biol. Rep.* 33: 1876-1892.

Byrne, O.M., Hardie, D.C., Khan, T.N., Speijers, J. and Yan, G. (2008) Genetic analysis of pod and seed resistance to pea weevil in a *Pisum sativum* × *P. fulvum* interspecific cross. *Aust. J. Agric. Res.* 59: 854– 862.

Canci, H. and Toker, C. (2009a) Evaluation of yield criteria for drought and heat resistance in chickpea (Cicer arietinum L.). *J. Agron. Crop Sci.* 195: 47–54.

Canci, H. and Toker, C. (2009b) Evaluation of annual wild Cicer species for drought and heat resistance under field conditions. *Gen. Res. Crop Evol.* 56: 1–6.

Choudhary, A.K., Sultana, R., Pratap, A., Nadarajan, N., and Jha, U.C. (2011) Breeding for abiotic stresses in pigeonpea. *J. Food Leg.*  24: 165-174.

Concibido, V.C., La Vallee, B., Mclaird, P., Pineda, N., Meyer, J., Hummel, L., et al. (2003) Introgression of a quantitative trait locus for yield from *Glycine soja* into commercial soybean cultivars. *Theor. Appl. Genet.* 106: 575–582.

Cortés, A.J., Monserrate, F.A., Ramírez-Villegas, J., Madriñán, S., and Blair, M.W. (2013) Drought tolerance in wild plant populations: the case of common beans (Phaseolus vulgaris L.). *PLoS One8*: e62898.

Coyne, C.J., Kumar, S., von Wettberg, E.J., Marques, E., Berger, J.D., Redden, R.J.,et al.(2020) Potential and limits of exploitation of crop wild relatives for pea, lentil, and chickpea improvement. *Legum. Sci.* *2*: e36.

Cruz Ruiz, S.A., Espitia, E., Mosquera Cifuentes, G.M., and Beebe, S.E. (2021) Using bean populations derived from *P. acutifolius* to advance toward generation of new bean varieties and discerning the traits and genetic base associated to heat tolerance.https://doi.org/10.7910/DVN/LX7PRK

Dalvi, V.A., Saxena, K.B., and Madrap, I.A. (2008) Fertility restoration in cytoplasmic-nuclear male-sterile lines derived from 3 wild relatives of pigeonpea. *J. Hered.* 99: 671-673.

Das, A., Saxena, S., Kumar, K., Tribhuvan, K.U., Singh, N.K., and Gaikwad, K. (2020) Non-coding RNAs having strong positive interaction with mRNAs reveal their regulatory nature during flowering in a wild relative of pigeonpea (*Cajanus scarabaeoides*). *Mol. Bio. Rep.* 47: 3305-3317.

Denwar, N.N., Simpson, C.E., Starr, J.L., Wheeler, T.A., and Burow, M.D. (2021) Evaluation and selection of interspecific lines of groundnut (*Arachis hypogaea L.*) for resistance to leaf spot disease and for yield improvement. *Plants* *10*: 873.

Diers, B.W., Keim, P., Fehr, W.R., and Shoemaker, R.C. (1992) Rflp analysis of soybean seed protein and oil content. *Theor. Appl. Genet.* 83: 608–612.

Dutra, W.F., Guerra, Y.L., Ramos, J.P., Fernandes, P.D., Silva, C.R., Bertioli, D.J. et al. (2018) Introgression of wild alleles into the tetraploid peanut crop to improve water use efficiency, earliness and yield. *PLoS One*13: e0198776.

Eker, T., Erler, F., Adak, A., Imrek, B., Guven, H., Tosun, H.S., et al. (2018) Screening of chickpea accessions for resistance against the pulse beetle, *Callosobruchus chinensis* L. (Coleoptera: Bruchidae). *J. Stored Prod. Res.* 76:51–57.

Fondevilla, S., and Rubiales, D. (2012) Powdery mildew control in pea.A review. *Agron. Sustain. Dev.*32: 401-409.

Golla, S.K., Rajasekhar, P., Akbar, S.M.D., Sharma, H.C.(2018) Proteolytic activity in the midgut of *Helicoverpaarmigera* (Noctuidae: Lepidoptera) larvae fed on wild relatives of Chickpea, *Cicer arietinum*. *J. Econ. Entomol.*111: 2409–2415.

Guimarães, P.M., Brasileiro, A.C., Morgante, C.V., Martins, A.C., Pappas,G., Silva, O. B., et al. (2012) Global transcriptome analysis of two wild relatives of peanut under drought and fungi infection. *BMC Genomics*13: 1-15.

Gupta, S. and Parihar, A.K. (2015) Broadening the genetic base of pulse crops. In: Dixit GP, Singh J, Singh NP (eds) Pulses- Challenges and opportunities under changing climate scenario. Indian Society of Pulses Research and Development, IIPR, Kanpur, pp 86–101

Infantino, A.,Porta-Puglia, A. and Singh, K.B. (1996) Screening wild *Cicer* species for resistance to *Fusarium* wilt. *Plant Dis.* 80: 42–44.

Kaur, L., Sandhu, J.S., Malhotra, R.S., Imtiaz, M., Sirari, A. (2012) Sources of stable resistance to Ascochyta blight in exotic kabulichickpea.*J. Food Legume.*25:79–80.

Khoury, C.K., Casta.eda-Alvarez, N.P., Achicanoy, H.A., Sosa, C.C., Bernau, V., Kassa, M.T., et al. (2015) Crop wild relativesof pigeonpea [*Cajanus cajan* (L.) Millsp.]: distributions,ex situ conservation status, and potential genetic resources forabiotic stress tolerance. *Biol. Conserv.*184: 259–270.

Knights, E.J., Southwell, R.J., Schwinghamer, M.W., Harden, S. (2008) Resistance to *Phytophthora medicaginis* Hansen and Maxwell in wild *Cicer* species and its use in breeding root rot resistant chickpea (*Cicer arietinum* L.). *Aust. J. Agric. Res.* 59: 383–387.

Kofsky, J., Zhang, H. and Song, B.H. (2018) The untapped genetic reservoir: The past, current, and future applications of the wild soybean (*Glycine soja*). *Front. Plant Sci.* 9:949

Koh, H.-M.,Seo, B.Y., Kim, K.H., Kim, J-M., Lee, T., Heo, J.,et al. (2020) Genetic analysis of foxglove aphid (*Aulacorthumsolani Kaltenbach*) resistance in soybeans.*Korean J. Breed. Sci.* 52: 354–361.

Kulkarni, N.K., Reddy, A.S., Kumar, P.L., Vijaynarasimha, J., Rangaswamy, K.T., Muniyappa, V., et al. (2003) Broad-based resistance to pigeonpea sterility mosaic disease in accessions of *Cajanus scarabaeoides* (L.) Benth. *Indian J. Plant Prot.* *31*: 6-11.

Kumar, P.L., Latha, T.K.S., Kulkarni, N.K., Raghavendra, N., Saxena, K.B., Waliyar, F., et al. (2005) Broad‐based resistance to pigeonpea sterility mosaic disease in wild relatives of pigeonpea (*Cajanus: Phaseoleae*). *Ann. Appl. Biol.* *146* : 371-379.

Kumar, S., Singh, M., Malhotra, N., Blair, M.W., Sharma, J.P., and Gupta, R. (2021) Introgression of anthracnose resistance into the background of locally adapted common bean landraces. *Euphytica*, 217: 1-11.

Leamy, L.J., Zhang, H., Li, C., Chen, C.Y. and Song, B.H. (2017) A genome-wide association study of seed composition traits in wild soybean (*Glycine soja*). *BMC Genomics*18 :18.

Lee, J.S., Yoo, M.H., Jung, J.K., Bilyeu, K.D., Lee, J. D., and Kang, S. (2015b) Detection of novel QTLs for foxglove aphid resistance in soybean. *Theor. Appl. Genet.* 128: 1481–1488.

Li, Y., Ruperao, P., Batley, J., Edwards, D., Davidson, J., Hobson, K., et al. (2017) Genome analysis identified novel candidate genes for Ascochyta blight resistance in chickpea using whole genome re-sequencing data. *Front. Plant Sci., 8*: 359.

Mahuku, G.S., Jara, C., Cajiao, C., and Beebe, S. (2003) Sources of resistance to angular leaf spot (*Phaeoisariopsisgriseola*) in common bean core collection, wild *Phaseolus vulgaris* and secondary gene pool. *Euphytica*130: 303-313.

Mahuku, G.S., Jara, C.E., Cajiao, C., and Beebe, S. (2002) Sources of resistance to *Colletotrichum lindemuthianum* in the secondary gene pool of *Phaseolus vulgaris* and in crosses of primary and secondary gene pools. *Plant Dis.* 86: 1383-1387.

Mallikarjuna, N., Jadhav, D.R., Reddy, M.V., and Dutta-Tawar, U. (2005) Introgression of Phytophthora blight disease resistance from *Cajanus platycarpus* into short duration pigeonpea [*Cajanus cajan* (L.) Millsp.]. *Indian J Genet.and Plant Breed.* 65: 261-263.

Maqbool, M.A., Aslam, M., and Ali, H. (2017) Breeding for improved drought tolerance in Chickpea (*Cicer arietinum* L.). *Plant Breed.* 136: 300–318.

Nay, M.M., Souza, T.L., Raatz, B., Mukankusi, C.M., Gonçalves-Vidigal, M.C., Abreu, A.F. et al. (2019) A review of angular leaf spot resistance in common bean. *Crop Sci*. 9:1376-1391.

Oki, N., Takagi, K., Ishimoto, M., Takahashi, M. and Takahashi, M. (2019) Evaluation of the resistance effect of QTLs derived from wild soybean (*Glycine soja*) to common cutworm (*Spodoptera lituraFabricius*). *Breed. Sci.* 69: 529-535.

Pande, S., and Rao, J. N. (2001) Resistance of wild Arachis species to late leaf spot and rust in green house trials. *Plant Dis.*85: 851-855.

Pande, S., Ramgopal, D., Kishore, G.K., Mallikarjuna, N., Sharma, M., Pathak, M. and Narayana, R.J. (2006) Evaluation of wild *Cicer* species for resistance to Ascochyta blight and Botrytis gray mold in controlled environment at ICRISAT, Patancheru, *India. Int Chickpea Pigeonpea Newsl.* 13: 25–27.

Pande, S., Sharma, M., Gaur, P.M. and Gowda, C.L.L. (2010) Host plant resistance to *Ascochyta* blight of chickpea.Information Bulletin No. 82. Patancheru 502 324.International Crops Research Institute for the Semi-Arid Tropics, Andhra Pradesh, p 40.

Podder, R., Banniza, S. and Vandenberg, A. (2013) Screening of wild and cultivated lentil germplasm for resistance to stemphylium blight. *Plant Genet.Resour.* 11: 26-35.

Prince, S.J., Song, L., Qiu, D., Maldonado Dos Santos, J.V., Chai, C., Joshi, T., et al. (2015) Genetic variants in root architecture-related genes in a *Glycine soja* accession, a potential resource to improve cultivated soybean. *BMC Genomics* 16: 132.

Qi, X., Li, M.W., Xie, M., Liu, X., Ni, M., Shao, G., et al. (2014) Identification of a novel salt tolerance gene in wild soybean by whole-genome sequencing. *Nat. Commun.* 5: 4340.

Ramakrishna, G., Kaur, P., Singh, A., Yadav, S.S., Sharma, S., Singh, N.K., et al. (2021) Comparative transcriptome analyses revealed different heat stress responses in pigeonpea (*Cajanus cajan*) and its crop wild relatives. *Plant Cell Rep.* *40*: 881-898.

Ramgopal, D., Srivastava, R., Pande, S., Rathore, A., Jadhav, D., Sharma, M., et al. (2013) Introgression of Botrytis grey mould resistance genes from Cicer reticulatum (bgmr1cr) and *C. echinospermum* (bgmr1ce) to chickpea (*C. arietinum*). *Plant Genet. Resour.*  *11*: 212-216.

Rani, A., Devi, P., Jha, U.C., Sharma, K.D., Siddique, K.H. and Nayyar, H. (2020) Developing climate-resilient chickpea involving physiological and molecular approaches with a focus on temperature and drought stresses. *Front. Plant Sci.* 10:1759.

Reddy, L.J., 1990. Pigeonpea: Morphology. In: Nene, Y.L., Hall, S.D. Sheila, V.K., (Eds.), The Pigeonpea. CAB International, Wallingford, UK. pp. 47-87.

Rodda, M.S., Davidson, J., Javid, M., Sudheesh, S., Blake, S., Forster, J.W., et al. (2017) Molecular breeding for ascochyta blight resistance in lentil: current progress and future directions. *Front. Plant Sci.* *8*: 1136.

Saeed, A. andDarvishzadeh, R. (2017) Association analysis of biotic and abiotic stresses resistance in chickpea (*Cicer* spp.) using AFLP markers. *Biotechnol. Biotechnol. Equip.* 31: 698–708.

Saxena, K.B., Ariyanayagam, R.P. and Reddy, L.J. (1992) Genetics of a high-selfing trait in pigeonpea. *Euphytica* 59: 125-127.

Sharma, H.C., Bhagwat, M.P., Pampapathy, G., Sharma, J.P. andRidsdill-Smith, T.J. (2006) Perennial wild relatives of chickpea as potential sources of resistance to *Helicoverpaarmigera.Genet. Resour. Crop Evol*. 53: 131-138.

Sharma, H.C., Sujana, G., and Manohar Rao, D. (2009) Morphological and chemical components of resistance to pod borer, Helicoverpaarmigera in wild relatives of pigeonpea. *Arthropod Plant Interact.* 3: 151-161.

Sharma, S. (2017) Prebreeding using wild species for genetic enhancement of grain legumes at ICRISAT. *Crop Sci*. 57: 1132-1144.

Sharma, S., and Upadhyaya, H.D. (2016) Pre-breeding to expand primary genepool through introgression of genes from wild Cajanus species for pigeonpea improvement. *Legume Perspectives* 11: 17-20.

Singh, M., Bisht, I.S., Dutta, M., Kumar, K., Basandrai, A.K., Kaur, L., et al. (2014) Characterization and evaluation of wild annual Cicer species for agro-morphological traits and major biotic stresses under northwestern Indian conditions.*Crop Sci* 54: 229-239.

Singh, M., Kumar, S., Basandrai, A. K., Basandrai, D., Malhotra, N., Saxena, D.R. et al. (2020) Evaluation and identification of wild lentil accessions for enhancing genetic gains of cultivated varieties. *PloS One*, *15*: e0229554.

Singh, S., Gumber, R. K., Joshi, N., and Singh, K. (2005) Introgression from wild *Cicer reticulatum* to cultivated chickpea for productivity and disease resistance. *Plant Breed*. 124: 477-480.

Singh, S.P. (2001) Broadening the genetic base of common bean cultivars: a review. *Crop Sci.* *41*: 1659-1675.

Sinha, P., Singh, V.K., Saxena, R.K., Khan, A.W., Abbai, R., Chitikineni, A., et al. (2020) Superior haplotypes for haplotype-based breeding for drought tolerance in pigeonpea (Cajanus cajan L.). *Plant Biotechnol. J.* 18: 2482-2490.

Smýkal, P., Aubert, G., Burstin, J., Coyne, C.J., Ellis, N.T., Flavell, A.J., et al. (2012) Pea (*Pisum sativum* L.) in the genomic era. *Agronomy* *2*: 74-115.

Souter, J.R., Gurusamy, V., Porch, T.G. and Bett, K.E. (2017) Successful introgression of abiotic stress tolerance from wild Tepary bean to common bean. *Crop Sci.* 57:1160-1171.

Subbarao, G. V. (1988). *Salinity tolerance in pigeonpea (Cajanus cajan (L.) Millsp.)and its wild relatives* (Doctoral dissertation, Indian Institute of Technology, Kharagpur, West Bengal).153.

Subrahmanyam, P., Anaidu, R., Reddy, L.J., Kumar, P.L. and Ferguson, M.E. (2001) Resistance to groundnut rosette disease in wild Arachis species. *Ann. Appl. Biol.139*: 45-50.

Subrahmanyam, P., Moss, J.P. and Rao, V.R. (1983) Resistance to peanut rust in wild Arachis species. *Plant Dis.* 67: 209-212.

Thompson, J., Reen, R., Clewett, T., Sheedy, J., Kelly, A., Gogel, B.and Knights, E. (2011) Hybridisation of Australian chickpea cultivars with wild *Cicer* spp. increases resistance to root-lesion nematodes (*Pratylenchusthornei* and *P. neglectus*). *Aust. Plant. Pathol.* 40: 601-611.

Toker, C., Berger, J., Eker, T., Sari, D., Sari, H., Gokturk, R.S., Kahraman, A. et al. (2021) *Cicer turcicum*: A new *Cicer* species and its potential to improve chickpea.*Front. Plant. Sci.* 12: 662891.

Tullu, A., Banniza, S., Tar’an, B., Warkentin, T., and Vandenberg, A. (2010) Sources of resistance to ascochyta blight in wild species of lentil (*Lens culinaris*Medik.). *Genet. Resour. Crop Evol.*57 :1053-1063.

Wang, X., Guo, R., Li, M., Liu, Y., Zhao, M., Fu, H., et al. (2019) Metabolomics reveals the drought-tolerance mechanism in wild soybean (*Glycine soja*). *Acta Physiol. Plant.* 41: 1-11.

Winter, P., Benko-Iseppon, A.-M.,Hüttel,B., Ratnaparkhe, M.,Tullu, A.,Sonnante, G., et al. (2000) A linkage map of the chickpea (*Cicer arietinum L*.) genome based on recombinant inbred lines from a *C. arietinum* × *C. reticulum* cross: Localization of resistance genes for *fusarium* wilt races 4 and 5*. Theor. Appl. Genet.*101: 1155–1163.

Ye, G., McNeil, D.L., and Hill, G.D. (2002) Breeding for resistance to lentil Ascochyta blight. *Plant Breed.* 121: 185-191.

Yuan. C.P., Wang, Y.J., Zhao, H.K., Zhang, L., Wang, Y.M., Liu, X.D., et al. (2016) Genetic diversity of rhg1 and Rhg4 loci in wild soybeans resistant to soybean cyst nematode race 3. *Genet. Mol. Res.* 10: 15.

Zhang, S., Zhang, Z., Bales, C., Gu, C., Difonzo, C., Li, M., et al. (2017a) Mapping novel aphid resistance QTL from wild soybean, *Glycine soja* 85-32. *Theor. Appl. Genet.* 130:1941–1952.

Zhang, S., Zhang, Z., Wen, Z., Gu, C., An, Y. C., Bales, C., et al. (2017b) Fine mapping of the soybean aphid-resistance genes Rag6 and Rag3c from *Glycine soja* 85-32. *Theor.Appl. Genet.* 130: 2601–2615.

Zhang, W., Liao, X., Cui, Y., Ma, W., Zhang, X., Du, H. et al. (2019) A cation diffusion facilitator, GmCDF1, negatively regulates salt tolerance in soybean. *PLoS Genet.* 15:e1007798.
